# A long-lived IL-2 mutein that selectively activates and expands regulatory T cells as a therapy for autoimmune disease

**DOI:** 10.1016/j.jaut.2018.10.017

**Published:** 2018-12

**Authors:** Laurence B. Peterson, Charles J.M. Bell, Sarah K. Howlett, Marcin L. Pekalski, Kevin Brady, Heather Hinton, Denise Sauter, John A. Todd, Pablo Umana, Oliver Ast, Inja Waldhauer, Anne Freimoser-Grundschober, Ekkehard Moessner, Christian Klein, Ralf J. Hosse, Linda S. Wicker

**Affiliations:** a Roche Innovation Center Basel, Roche Pharma Research & Early Development (pRED), Grenzacherstrasse 124, 4070, Basel, Switzerland; b JDRF/Wellcome Diabetes and Inflammation Laboratory, Wellcome Centre for Human Genetics, Nuffield Department of Medicine, NIHR Oxford Biomedical Research Centre, University of Oxford, Oxford, UK; c JDRF/Wellcome Diabetes and Inflammation Laboratory, Department of Medical Genetics, Cambridge Institute for Medical Research, NIHR Cambridge Biomedical Research Centre, University of Cambridge, Cambridge, CB2 OXY, UK; d Roche Innovation Center Zurich, Roche Pharma Research & Early Development (pRED), Wagistrasse 10, 8952, Schlieren, Switzerland

**Keywords:** Autoimmunity, T_reg_ expansion, IL-2 mutein, Cytokine therapy, Immunotherapy

## Abstract

Susceptibility to multiple autoimmune diseases is associated with common gene polymorphisms influencing IL-2 signaling and T_reg_ function, making T_reg_-specific expansion by IL-2 a compelling therapeutic approach to treatment. As an *in vivo* IL-2 half-life enhancer we used a non-targeted, effector-function-silent human IgG1 as a fusion protein. An IL-2 mutein (N88D) with reduced binding to the intermediate affinity IL-2Rβγ receptor was engineered with a stoichiometry of two IL-2N88D molecules per IgG, *i.e.* IgG-(IL-2N88D)_2_. The reduced affinity of IgG-(IL-2N88D)_2_ for the IL-2Rβγ receptor resulted in a T_reg_-selective molecule in human whole blood pSTAT5 assays. Treatment of cynomolgus monkeys with single low doses of IgG-(IL-2N88D)_2_ induced sustained preferential activation of T_regs_ accompanied by a corresponding 10–14-fold increase in CD4^+^ and CD8^+^ CD25^+^FOXP3^+^ T_regs_; conditions that had no effect on CD4^+^ or CD8^+^ memory effector T cells. The expanded cynomolgus T_regs_ had demethylated *FOXP3* and *CTLA4* epigenetic signatures characteristic of functionally suppressive cells. Humanized mice had similar selective *in vivo* responses; IgG-(IL-2N88D)_2_ increased T_regs_ while wild-type IgG-IL-2 increased NK cells in addition to T_regs_. The expanded human T_regs_ had demethylated *FOXP3* and *CTLA4* signatures and were immunosuppressive. These results describe a next-generation immunotherapy using a long-lived and T_reg_-selective IL-2 that activates and expands functional T_regs_*in vivo*. Patients should benefit from restored immune homeostasis in a personalized fashion to the extent that their autoimmune disease condition dictates opening up the possibility for remissions and cures.

## Highlights

• A human IL-2 molecule mutated to decrease binding to the intermediate affinity IL-2 receptor preferentially activates Tregs.
• Two IL-2 muteins fused to human IgG1 allow for sustained, preferential expansion of Tregs in cynomolgus and humanized mice.
• As compared to the wild type IL-2 fusion protein, humanized mice expand fewer NK cells in response to the mutein.
• The dynamic range of Treg increase based on dose suggests the ability to individualize dosing for particular diseases.

## 1 Introduction

Interleukin-2 (IL-2), a molecule critical for immune homeostasis, both promotes and regulates immune responses to foreign antigens as well as to naturally occurring self-antigens [1,2]. IL-2 plays a key role in immune tolerance to “self” through its effects on the maintenance of T_regs_ that control self-reactive effector T cells that have escaped deletion in the thymus [3,4]. Genetic studies in autoimmune disease identified polymorphisms in IL-2 pathway genes encoding IL-2RA (CD25), IL-2, CTLA-4 and PTPN2 as key drivers in autoimmune disease susceptibility [[5], [6], [7], [8], [9], [10]]. Consistent with these genetic studies, functional studies demonstrated diminished effects of T_regs_ *in vitro* and *in vivo*. T_reg_ deficiencies in autoimmune disorders have been variously ascribed to a decrease in IL-2 production or a decrease in response to IL-2 leading to a diminution in T_reg_ numbers and/or a reduction of T_reg_ functional activity [[11], [12], [13], [14], [15]]. The sum of these studies suggests a therapeutic paradigm of *one pathway treating many autoimmune or inflammatory diseases* might be possible with T_reg_ adoptive transfers [[16], [17], [18]] or engineering a pharmacologically effective and T_reg_-specific IL-2 [19,20].

An overarching phenotype to specifically identify and characterize T_regs_ beyond CD25^+^, FOXP3^+^ and CD127^−^ remains elusive. However, new studies continue to identify incremental improvements in markers such as TIGIT, CD226, CD15s, CCR4 and FCRL3, allowing better discrimination between functionally suppressive T_regs_ and other immune cells [[21], [22], [23], [24]]. The expression of the transcription factor FOXP3 has been a hallmark of T_reg_ identification but its specificity was questioned when it was found that in humans, activated CD4^+^ and CD8^+^ effector T cells can express FOXP3 [25]. More recently it was shown that only functional T_regs_, and not activated CD4^+^ effector cells, have a fully demethylated epigenetic signature in a conserved region of intron 1 in *FOXP3* termed the TSDR (T_reg_-specific demethylated region) [26,27]. The exclusivity of this *FOXP3* TSDR demethylated signature in addition to a fully demethylated epigenetic signature in exon 2 of *CTLA4* [28,29] has advanced our ability to identify *bona fide* functional T_regs_. In addition to the more frequently studied CD4^+^ T_regs_, a CD8^+^ T_reg_ subset expressing CD25 and FOXP3 has been identified in humans treated with anti-CD3, mice undergoing allogeneic bone marrow transplantation and both humans and mice treated with low-dose recombinant human IL-2 (Proleukin^®^, aldesleukin) [[30], [31], [32], [33]]; these CD8^+^ T_regs_ were functionally suppressive *in vitro* and *in vivo*. With the clinical success of Proleukin in chronic graft versus host disease (GVHD) [34,35], a therapy that expands both CD4^+^ and CD8^+^ T_regs_ may increase the number of patients that achieve a clinical benefit in this severe immune-based disease [36].

In this decade, the trend in approved new molecular entities (NMEs) has increased for autoimmune diseases with biologics accounting for much of this growth [37,38]. Despite this surge, there is still an unmet need. In rheumatoid arthritis (RA), where the most success has occurred, 60% of moderate-to-severe RA patients still remain classified as inadequate responders and an ACR70 response (70% or greater improvement) is unlikely in all but 10% [39]. Despite all of the NMEs studied in type 1 diabetes, insulin is still the only treatment [40,41]. Clear progress has been made but large unmet medical needs still exist for most patients suffering from inflammatory and autoimmune disorders. However, recent evidence suggests there is potential for this to change. A recent case study showed that low-dose Proleukin had dramatic effects in a refractory moderate-to-severe systemic lupus erythematosus (SLE) patient [42]. Reduced disease activity was also observed in a low-dose Proleukin interventional study in 38 moderate-to-severe SLE patients [43].

In this study, we describe our efforts to engineer a pharmacologically superior and T_reg_-selective human IL-2 for the treatment of autoimmunity and other immune-based disorders. We aimed for an IL-2 variant that preferentially binds and activates cells expressing high levels of the IL-2Rαβγ receptor over those cells signaling primarily through the β and γ receptor chains. This can be achieved by either increasing IL-2 affinity to the alpha chain, which was explored already by others [19], or decreasing IL-2 affinity to the beta chain. Taking the latter approach, we developed an IL-2 mutein having a 30–80-fold reduced ability to activate IL-2 receptors present on CD4^+^ and CD8^+^ effector T cells and NK cells, which predominately signal through the intermediate affinity form of the receptor (IL-2Rβγ), with only a minimal reduction in its ability (6-fold) to activate high affinity IL-2Rαβγ receptors present at the highest levels on T_regs_. This IL-2 mutein was coupled to an effector-silent human IgG1 in a 2:1 stoichiometry to enhance its pharmacologic half-life and enhance its avidity to T_reg_ high-affinity IL-2Rαβγ receptors. This new IL-2 therapeutic was evaluated *in vitro* in a human whole blood pSTAT5 assay and *in vivo* in cynomolgus monkeys and humanized mice; under all testing conditions, the new molecule was highly T_reg_-selective. Its administration *in vivo* activated and expanded CD4^+^ and CD8^+^ CD25^+^FOXP3^+^ T_regs_ with epigenetic signatures at *FOXP3* and *CTLA4* of functional immunosuppressive T_regs_. Based on these enhanced and selective T_reg_ responses, we think this future therapeutic has the potential to restore the immune homeostasis that is perturbed in most autoimmune diseases.

## 2 Results

### 2.1 Reduced binding of IgG-(IL-2N88D)_2_ to IL-2Rβγ

**Fig. 1:**
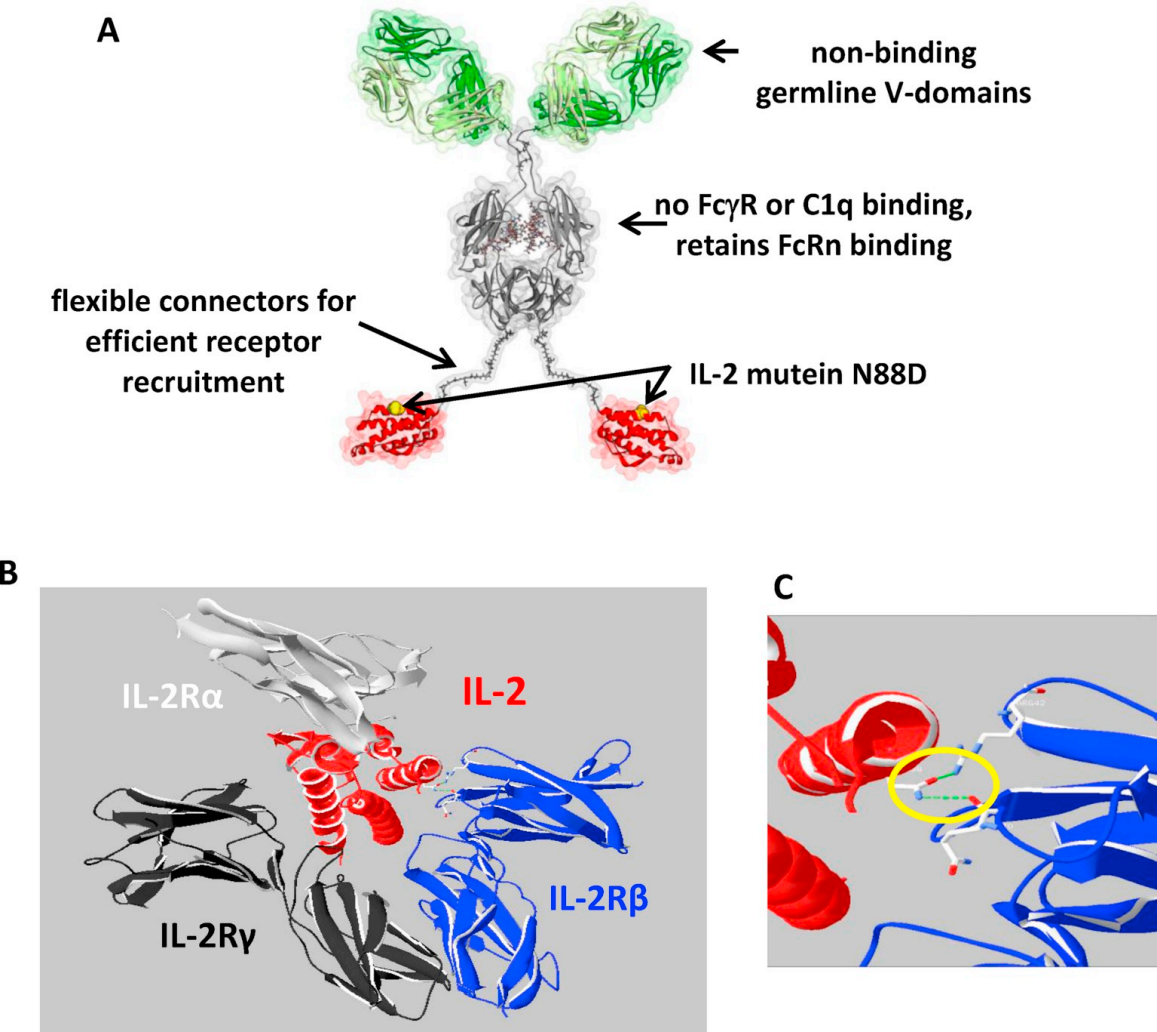
**The IgG-IL-2 fusion protein with the IL-2N88D mutein.** (**A**) The IgG-(IL-2N88D)_2_ fusion protein is shown schematically; the N88D point mutation is yellow. (**B**) Ribbon diagrams of wild-type human IL-2 (depicted in red) with its high affinity IL-2Rαβγ receptor (derived from the crystal structure (pdb code 2b5i) obtained by Wang et al. [44]). The chains of the alpha, beta and gamma receptors are shown in silver, blue, and black. Asn88 is shown in space filling representation. (**C**) Expanded view of the interaction of wild type IL-2 (asparagine 88) with IL-2Rβ.

By substituting aspartic acid (D) for asparagine (N) at position 88 in human IL-2, we engineered a novel long-lived bivalent fusion protein IgG-(IL-2N88D)_2_ (Fig. 1A) with reduced binding to the intermediate affinity IL-2Rβγ receptor, more precisely, to the β-chain of the receptor complex [44]. Ribbon diagrams of IL-2 and its high affinity trimeric receptor illustrate the nominal binding of IL-2 to IL-2Rαβγ (Fig. 1B) and in particular asparagine 88 of IL-2 to IL-2Rβ (Fig. 1C). We quantified the binding interactions of human IgG-(IL-2N88D)_2_ to IL-2Rβγ (Table 1) and IL-2Rα (Supplemental Table 1) receptors of human and cynomolgus and compared them to those previously acquired with wild-type human IL-2 fusion proteins [29]. Comparable association rates (k_a_) were seen to human and cynomolgus IL-2Rβγ regardless of the IL-2 fusion protein tested. In contrast, the dissociation rates (k_d_) of IgG-(IL-2N88D)_2_ were faster than either of the wild-type molecules on both species of IL-2Rβγ. The faster dissociation rates of IgG-(IL-2N88D)_2_ reduced the binding affinities (K_D_) to human (240 pM) and cynomolgus (570 pM) IL-2Rβγ receptors compared to wild-type IgG-IL-2 (40 and 180 pM, respectively) and IgG-(IL-2)_2_ (3 and 40 pM, respectively). The N88D point mutation had no effect on binding to the IL-2Rα chain and comparable steady state K_D_ results were seen for all IL-2 molecules tested.

**Table 1:** **Measuring the binding of human IL-2 fusion proteins to the IL-2Rβγ receptor**. The association (k_a_) and dissociation (k_d_) rate constants and ‘apparent’ binding affinities (K_D_) for three different IL-2 fusion proteins were determined by surface plasmon resonance on a BIACORE T200 by applying a globally fitted 1:1 interaction model for kinetic analyses.

Human	Species of IL-2Rβγ	k_a_ (1/Ms)	k_d_ (1/s)	K_D_ apparent (pM)
IgG-(IL-2N88D)_2_	human cynomolgus	1.28 × 10^6^ 0.80 × 10^6^	310 × 10^−6^ 450 × 10^−6^	240 570
IgG-IL-2	human cynomolgus	1.0 × 10^6^ 0.62 × 10^6^	40 × 10^−6^ 110 × 10^−6^	40 180
IgG-(IL-2)_2_	human cynomolgus	2.4 × 10^6^ 1.4 × 10^6^	8 × 10^−6^ 53 × 10^−6^	3 40

### 2.2 pSTAT responses are reduced in cells lacking the high affinity IL-2 receptor

**Fig. 2:**
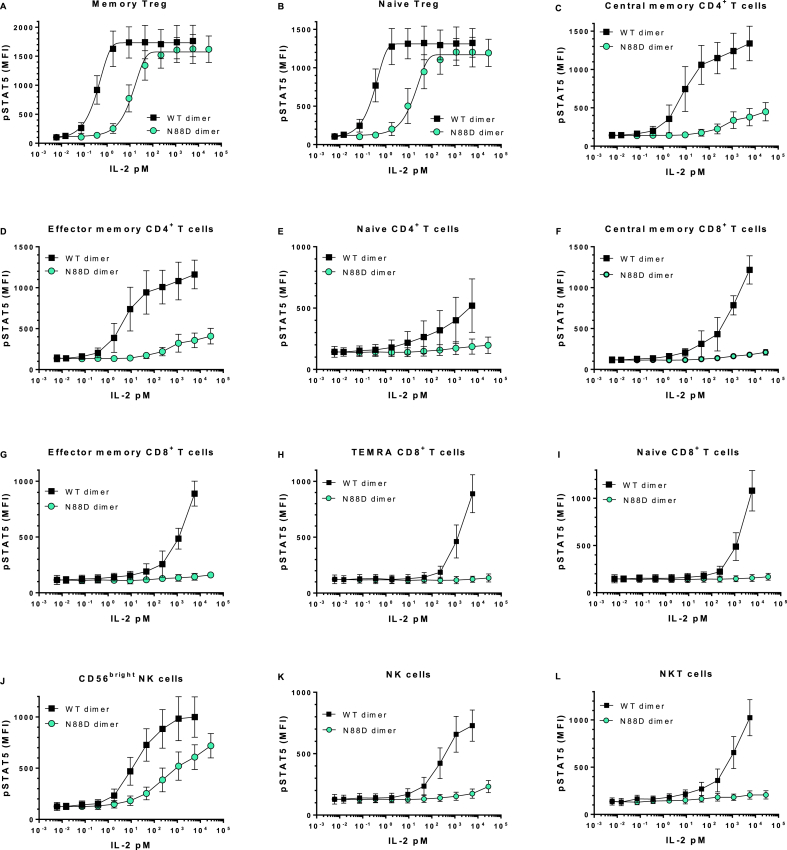
**IgG-(IL-2N88D)**_**2**_**demonstrates increased T**_**reg**_**selectivity compared to IgG-(IL-2)**_**2**_**in human whole blood pSTAT5 responses**. Wild type dimer IgG-(IL-2)_2_ and N88D dimer IgG-(IL-2N88D)_2_ were tested for their ability to induce phosphorylation of STAT5 (pSTAT5) in human whole blood. TEMRA CD8^+^ T cells refer to terminally-differentiated memory T cells that have upregulated CD45RA (CD62L^−^, CD45RA^+^). Results are shown for both IgG-(IL-2)_2_ and IgG-(IL-2N88D)_2_ from the same n = 10 donors and results are shown as the mean ± SD. See Supplemental Table 3 for antibody panel used and Supplemental Fig. 1 for the definition of cell subsets.

**Fig. 3:**
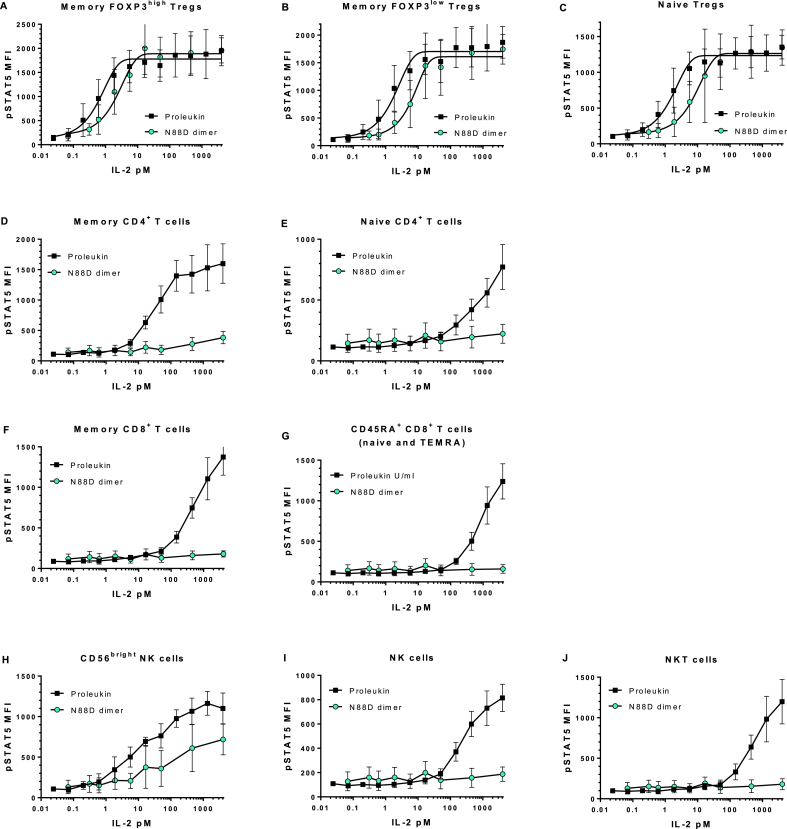
**Human whole blood pSTAT5 responses with IL-2**. Proleukin and IgG-(IL-2N88D)_2_ were tested for their abilities to induce pSTAT5 in human whole blood; n = 4 for Proleukin and n = 5 for IgG-(IL-2N88D)_2_. One of the donors was tested with Proleukin and IgG-(IL-2N88D)_2_ in the same test. None of the donors are the same as those in Fig. 2. See Supplemental Table 3 for the antibody panel used and Supplemental Fig. 2 for the definition of cell subsets.

Having determined that the binding affinity to the intermediate affinity receptor IL-2Rβγ was reduced, we compared the functional activity of IgG-(IL-2N88D)_2_ to a comparable fusion protein having wild-type IL-2, *i.e.* IgG-(IL-2)_2_, whose properties we described previously [29], as well as recombinant human IL-2, *i.e*. Proleukin (aldesleukin), in a human whole blood assay measuring STAT5 phosphorylation in cell types known to have differing levels of the high and intermediate forms of the IL-2 receptor [4]. All donors' memory and naïve T_regs_ that express high levels of the high affinity IL-2Rαβγ receptor responded to IgG-(IL-2N88D)_2_, IgG-(IL-2)_2_, and Proleukin with robust dose-dependent increases in pSTAT5 (Fig. 2A and B; Fig. 3A–C). The EC_50_s for pSTAT5 induction in memory and naïve T_regs_ were each 2 pM for Proleukin and 11 and 18 pM for IgG-(IL-2N88D)_2_ (Table 2). The 6–9-fold loss of potency on T_regs_ by IgG-(IL-2N88D)_2_ compared to Proleukin is consistent with its 6-fold reduction in binding affinity to IL-2Rβγ compared to IgG-IL-2 (monomeric IL-2) (Table 1) and comparable binding affinity for the IL-2Rα component of the high affinity receptor (Supplemental Table 1). Also consistent with its increased affinity to IL-2Rβγ as compared to monomeric IL-2 (Table 2), IgG-(IL-2)_2_ had an EC_50_ for pSTAT5 induction in memory and naïve T_regs_ of 0.3 pM. Interestingly, in all donors, the maximal pSTAT5 responses of naïve T_regs_ were only 60–70% of their memory T_regs_ suggesting that either a lower level of STAT5 is present to be phosphorylated or that there is a higher level of negative regulators of cytokine signaling in naïve T_regs_ as compared to memory T_regs_.

**Table 2:** **Human whole blood EC50s from pSTAT5 assay.** EC_50_s from various cell types were determined for Proleukin and IgG-(IL-2N88D)_2_ from the dose response titration of their abilities to induce the phosphorylation of STAT5 in human whole blood; (n = 10 donors, mean EC_50_ in pM).

HUMAN	Proleukin EC_50_ (pM)	IgG-(IL-2N88D)_2_ EC_50_ (pM)	Fold-change in EC_50_
CD4^+^ memory T_regs_	2	11	6-fold
CD4^+^ naïve T_regs_	2	18	9-fold
CD4^+^ effector memory T cells	29	>10,000	>300-fold
CD56^bright^ NK cells	>10	>1000	>100-fold

Proleukin stimulated strong pSTAT5 responses in CD4^+^ memory T cells (Fig. 3D) and wild type IgG-(IL-2)_2_ stimulated strong pSTAT5 responses in both central and effector memory subsets of CD4^+^ memory T cells (Fig. 2C and D), with an EC_50_ of less than 30 pM while IgG-(IL-2N88D)_2_ had little effect even at doses ≥1000 pM.

CD8^+^ memory T cells and their three subsets (central, effector and TEMRA), naïve CD4^+^ and CD8^+^ T cells, NKT cells and NK cells responded to IgG-(IL-2)_2_, and Proleukin but only at doses ≥100 pM (Fig. 2 E-I, K, L, Fig. 3E–G, I, J). All of these cell types were almost completely unresponsive to IgG-(IL-2N88D)_2_. Human CD56^bright^ NK cells, which constitutively express low levels of the high affinity IL-2 receptor, had an EC_50_ > 10 pM for both Proleukin and wild type IgG-(IL-2)_2_ however CD56^bright^ NK cells were >100-fold less responsive to IgG-(IL-2N88D)_2_ (Fig. 2, Fig. 3H) with an EC_50_ > 1000 pM (Table 2).

A limited set of pSTAT5 assays were done in cynomolgus whole blood with IgG-(IL-2N88D)_2_ and its more potent analogue wild-type IgG-(IL-2)_2_. Both memory and naïve CD4^+^ T_regs_ responded to IgG-(IL-2N88D)_2_ with robust dose-dependent pSTAT5 induction (Supplemental Fig. 3A–B) and EC_50_s were 13–20-fold higher than those of IgG-(IL-2)_2_ (Supplemental Table 2). As we saw in human blood, naïve T_reg_ pSTAT5 responses were less than those of memory T_regs_.

Distinct from humans whose CD4^+^ memory effector T cells are 70–80% CD25^+^ [45], only 20% of cynomolgus CD4^+^ memory effector T cells are CD25^+^ [29] and for this reason they were divided into CD25^−^ and CD25^+^ subsets for analysis. The CD25^−^ memory effector T cells were unresponsive to IgG-(IL-2N88D)_2_ and an EC_50_ could not be determined (Supplemental Fig. 3C). The CD25^+^ cells, while not unresponsive to IgG-(IL-2N88D)_2_, required doses >100 pM to induce pSTAT5 and an EC_50_ could not be determined (Supplemental Fig. 3D).

### 2.3 Pharmacodynamic biomarkers of IgG-(IL-2N88D)_2_ in nonhuman primates

So far, IgG-(IL-2N88D)_2_ had shown the *in vitro* characteristics we sought for a new class of immunotherapy: it had less binding affinity to IL-2Rβγ and was highly T_reg_-selective in whole blood pSTAT5 assays. Subsequent preclinical tests with IgG-(IL-2N88D)_2_ in cynomolgus monkeys used preset primary and secondary biomarkers. The primary biomarker was quantifying the *in vivo* expansion of CD4^+^CD25^hi^FOXP3^+^ T_regs_ as both the percentage of CD4^+^ T cells and as the absolute number of T_regs_/ml of blood. Secondary biomarkers consisted of (i) quantifying CD8^+^CD25^hi^FOXP3^+^ T_regs_, (ii) evaluating epigenetic methylation signatures of *FOXP3* and *CTLA4* in sorted cell subsets, (iii) quantifying the selectivity of activation markers pSTAT5, CD25, Ki-67 and FOXP3, and lastly, (iv) assessing the selectivity of cell expansion, *i.e.* T_regs_ versus CD4^+^ and CD8^+^ memory effector T cells, NK cells and eosinophils.

### 2.4 Robust *in vivo* expansion of CD4^+^ T_regs_ in response to IgG-(IL-2N88D)_2_

**Fig. 4:**
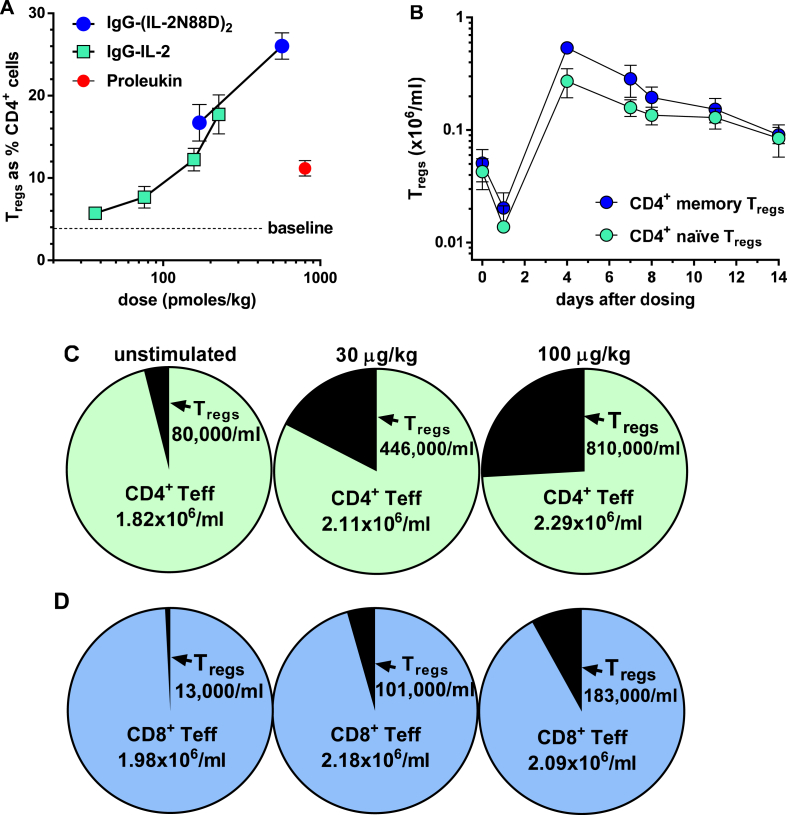
**Increase in cynomolgus T**_**regs**_**after IL-2 treatment.** (**A**) Effect of single injections of IgG-(IL-2N88D)_2_ and wild type monomer IgG-IL-2 and seven injections of Proleukin (800 pmol/kg, 3 times weekly) on the maximal expansion of total T_regs_ as the % CD4^+^ cells; n = 4–6 per dose, mean ± SE. The Proleukin results and the lower three doses of IgG-IL-2 have been published previously (29) and are shown here for comparison. (**B**) Time course of memory and naïve CD4^+^CD25^hi^FOXP3^+^ T_regs_ (x10^6^/ml) after 100 μg/kg (570 pmol/kg) of IgG-(IL-2N88D)_2_, n = 4, mean ± SD. (**C**) Number of CD4^+^CD25^hi^FOXP3^+^ T_regs_ and CD4^+^ effector T cells before (unstimulated) and after 100 μg/kg IgG-(IL-2N88D)_2_; means of n = 4. (**D**) Number of CD8^+^CD25^hi^FOXP3^+^ T_regs_ and CD8^+^ effector T cells before and after 100 μg/kg IgG-(IL-2N88D)_2_; means of n = 4.

Single *in vivo* doses of IgG-(IL-2N88D)_2_ induced substantial expansion of cynomolgus CD4^+^CD25^hi^FOXP3^+^ T_regs_ and are shown compared to the effects of multiple high doses of Proleukin or single low doses of wild-type IgG-IL-2 (Fig. 4A). Maximal responses to IgG-(IL-2N88D)_2_ occurred four days after dosing when T_regs_ expanded to 17% of the total CD4^+^ T cells at 170 pmol/kg (30 μg/kg) and 26% of total CD4^+^ T cells at 570 pmol/kg (100 μg/kg) (Fig. 4A). Wild type monomeric IgG-IL-2 was also a potent stimulator of cynomolgus T_regs_ while multiple high dose Proleukin induced more modest T_reg_ responses.

The time-dependent nature of T_reg_ expansion is shown as number of T_regs_/ml of blood for the 100 μg/kg dose (Fig. 4B). Both memory and naïve CD4^+^ T_regs_ fell in number 24 hours after treatment, reached maximal levels at four days, and were still 2–3-fold over baseline at 11 and 14 days. We have also observed a lymphocytopenia and rebound at 24–48 hours after Proleukin treatment in both humans and cynos [29]. This most likely reflects migration to, or retention in, tissues and secondary lymphoid tissues followed by a rebound to normal numbers.

The increase in the absolute number/ml of T_regs_ in blood was dose dependent as shown by the maximal increase after *in vivo* dosing (Fig. 4C). Before dosing with IgG-(IL-2N88D)_2_, cynomolgus CD4^+^ T_regs_ averaged 80,000/ml; after a 30 μg/kg dose, they increased 5.6-fold to 446,000/ml, and after a 100 μg/kg dose, they increased 10-fold to 810,000/ml. During this time of robust T_reg_ enhancement, CD4^+^ effector T cells remained in normal ranges.

### 2.5 IgG-(IL-2N88D)_2_ induces the *in vivo* expansion of cynomolgus CD8^+^ T_regs_

CD8^+^CD25^hi^FOXP3^+^ T_regs_ were monitored in cynomolgus as a secondary metric and averaged only 13,000/ml before treatment (Fig. 4D, Supplemental Fig. 4A), displayed a naïve phenotype (CD45RA^**+**^) and were not in cell cycle (Ki-67^−^) (Supplemental Fig. 4C). These low-abundance T_regs_ increased 8–14-fold after IgG-(IL-2N88D)_2_ injection reaching 101,000/ml after 30 μg/kg and 183,000/ml after 100 μg/kg (Fig. 4D, Supplemental Fig. 4B). These newly expanded CD8^+^CD25^hi^FOXP3^+^ T_regs_ were almost all memory cells (CD45RA^**−**^**)**, most of which were in cell cycle (Ki-67^+^) (Supplemental Fig. 4D). The overall increase in CD8^+^ cells was accounted for by this dramatic expansion of CD8^+^ T_regs_ and not by CD8^+^ T effector cells.

### 2.6 FOXP3 and CTLA4 epigenetic signatures are maintained in T_regs_ expanded *in vivo*

A demethylated signature of the *FOXP3* TSDR in intron 1 is a strong predictive indicator that T_regs_ are functional and immunosuppressive [27]. In contrast, activated human effector T cells can be induced to express FOXP3 protein but their *FOXP3* TSDR remains methylated and the cells are not immunosuppressive.

As another secondary *in vivo* metric, we looked for epigenetic changes occurring as a consequence of IgG-(IL-2N88D)_2_ treatment. We sorted CD4^+^CD25^hi^FOXP3^+^ T_regs_, naïve and memory effector CD4^+^ T cells and CD8^+^FOXP3^+^ T_regs_. Using next-generation sequencing of bisulfite-treated DNA, we quantified the methylation status of the ten CpG sites in the cynomolgus TSDR region of intron 1 of *FOXP3* and the nine CpG sites in cynomolgus exon 2 of *CTLA4*, a gene strongly associated with T_reg_ function [46,47].

**Fig. 5:**
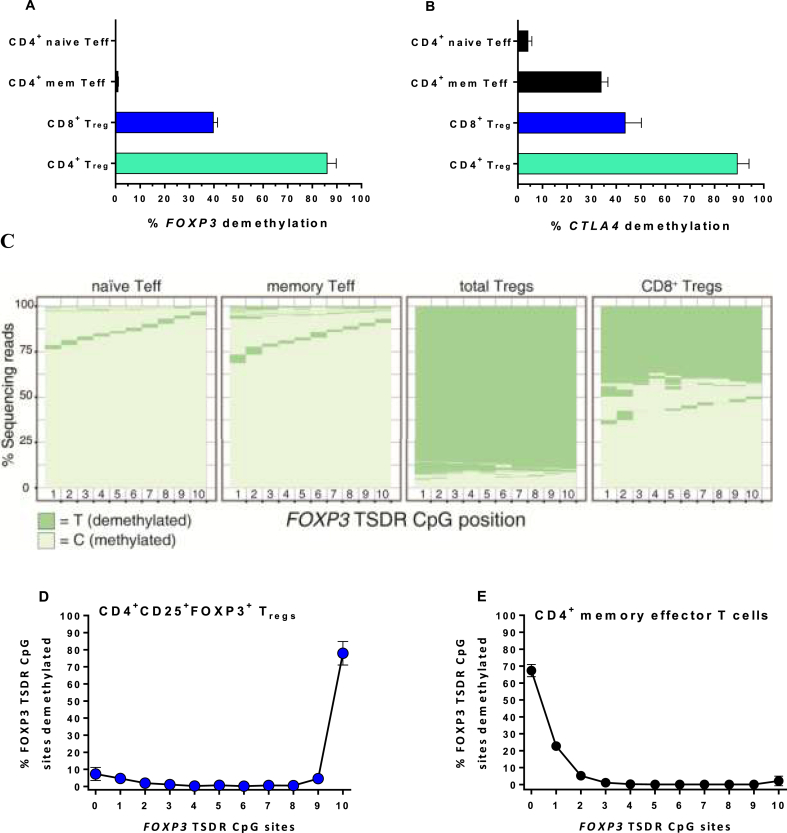
**Cynomolgus epigenetic signatures of *FOXP3* and *CTLA4***. The percentage of sequencing reads demethylated at 9 or 10 of the 10 CpG sites assessed in intron 1 of *FOXP3***(A)** and 8 or 9 of the 9 CpG sites assessed in exon 2 of *CTLA4* (**B**) in sorted cell subsets after T_reg_ expansion following IgG-(IL-2N88D)_2_ are shown as the mean ± SD, n = 4, 100 μg/kg. (**C**) Representative examples of methylated and demethylated sequencing reads at each CpG site for *FOXP3* in sorted cell subsets. See Supplemental Fig. 5 for an example of *CTLA4*. Representative examples of the percentage of reads demethylated at the number of *FOXP3* CpG sites indicated for CD4^+^ T_regs_ (**D**) and CD4^+^ memory effector T cells (**E**).

Following IgG-(IL-2N88D)_2_ treatment, *FOXP3* TSDR demethylation averaged 86% for CD4^+^CD25^hi^FOXP3^+^ T_regs_ (Fig. 5A), a strong predictor that these newly expanded T_regs_ are functionally immunosuppressive. In contrast, only a portion of CD8^+^FOXP3^+^ T_regs_ had a demethylated *FOXP3* signature, half that of the CD4^+^ T_regs_. In retrospect, CD8^+^FOXP3^+^ cells did not have CD25^hi^ expression included in the strategy used for sorting which may have contributed to a lower proportion of CD8^+^ T_regs_ appearing demethylated at the *FOXP3* TSDR (Fig. 5A). Whether this reduced the extent of demethylation reflects their natural state, remains for further testing. Lastly, CD4^+^ memory effector T cells were 2.7% demethylated at *FOXP3* and CD4^+^ naïve effector T cells were 0.1% demethylated, neither of which was indicative of a suppressive phenotype.

Quantifying *CTLA4* demethylation in this same set of sorted cells showed 89% for CD4^+^ T_regs_, 44% for CD8^+^ T_regs_, 34% with CD4^+^ memory effector T cells, and 4% for CD4^+^ naïve effector T cells (Fig. 5B). The sorting strategy for the CD8^+^FOXP3^+^ T_regs_ may again have played a role in their reduced level of *CTLA4* demethylation. Because CD4^+^ memory effector T cells have been previously induced to proliferate in response to antigen and have expressed CTLA-4 following such activation, it is likely that their pattern of *CTLA4* demethylation reflects the fact that the locus is poised for further expression.

As a means to visualize the epigenetic signatures of *FOXP3* and *CTLA4*, the results are graphed as the percentage of the total sequencing reads showing the status of each CpG site: methylated (C, cytosine) versus demethylated (T, thymine) (Fig. 5C, Supplemental Fig. 5). Additional information gained from these analyses showed that for CD4^+^ T_regs_ >82% of the *FOXP3* sequencing reads had 10 of 10 CpG sites demethylated (Fig. 5D) while CD4^+^ memory effector T cells had the opposite pattern, >95% of the *FOXP3* sequencing reads had none or only one of the CpG sites demethylated (Fig. 5E).

### 2.7 Ex vivo cell activation biomarkers correlate with IgG-(IL-2N88D)_2_ dosing

**Fig. 6:**
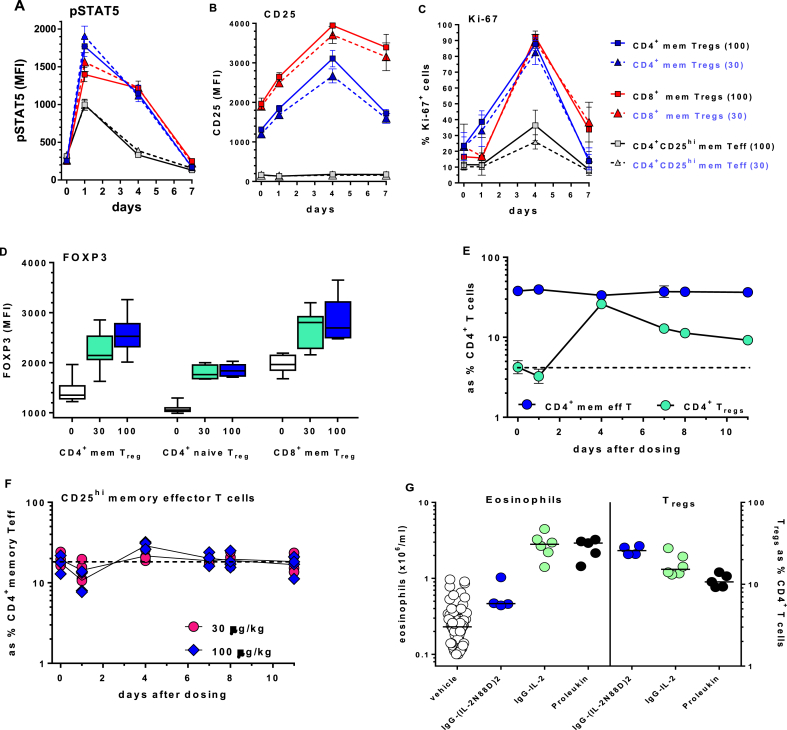
**Evaluation of pharmacodynamic biomarkers in cynomolgus**. *In vivo* dose and time-dependent changes in IgG-(IL-2N88D)_2_-induced (**A**) pSTAT5, (**B**) CD25 and (**C**) % of cells Ki-67^+^; ■ = 100 μg/kg, ▲ = 30 μg/kg. (**D**) Maximal dose-dependent changes in FOXP3 at 30 and 100 μg/kg. (**E**) Time-dependent changes in CD4^+^ T_regs_ compared to total CD4^+^ memory effector T cells; 100 μg/kg dose. Results in **A-E** are shown as the mean ± SD, n = 4. (**F**) Time-dependent changes in CD4^+^CD25^+^ memory effector T cells; individual animals (n = 4). (**G**) Effects IgG-(IL-2N88D)_2_ (100 μg/kg), wild type IgG-IL-2 (36 μg/kg) and Proleukin (see dosing regimen detailed in Fig. 4A) and the effects on eosinophils and T_regs_; each symbol represents an individual animal. Proleukin results were published previously (29) and are shown for comparison.

Other biomarkers we assessed were indicators of cell activation, *i.e*. pSTAT5, CD25, Ki-67 and FOXP3. Phosphorylation of STAT5 is a sensitive intracellular marker for IL-2-induced activation and can be accurately quantified *ex vivo* in whole blood [29]. After dosing with IgG-(IL-2N88D)_2_, memory T_regs_ were the most responsive cells in blood and their increase in pSTAT5 was seen out to four days after dosing (Fig. 6A). The CD25^+^ subset of CD4^+^ memory effector T cells produced a transient pSTAT5 response seen at day one. Little or no pSTAT5 induction was seen in any of the other cell types tested (Supplemental Fig. 6A).

Cell surface CD25 is an inducible biomarker whose expression levels correlate with *in vivo* IL-2 exposure [29]. After IgG-(IL-2N88D)_2_ administration, CD25 increased on CD4^+^ and CD8^+^ T_regs_ but not on CD4^+^CD25^+^ memory effector T cells despite their transient pSTAT5 response (Fig. 6B). Little or no CD25 expression was induced in the other cells tested (Supplemental Fig. 6B).

Upon activation, cells transit from G_0_/G_1_ into cell cycle and show a corresponding increase in intracellular Ki-67 [48]. After IgG-(IL-2N88D)_2_ treatment, movement into cell cycle was seen as early as day one in CD4^+^ T_regs_ with 80–92% of the T_regs_ Ki-67^+^ at 4 days (Fig. 6C). Other cells appeared to enter cell cycle, notably CD4^+^CD25^+^ memory effector T cells (Fig. 6C) and CD8^+^ memory effector T cells (Supplemental Fig. 6C) but neither subset showed increases in number (Fig. 4C, D).

Intracellular FOXP3, a hallmark of T_regs_, increased after IgG-(IL-2N88D)_2_ dosing in memory and naïve CD4^+^ T_regs_ and in memory CD8^+^ T_regs_ (Fig. 6D). Within each T_reg_ subset, the increases in FOXP3 were different from baseline (p ≤ 0.0006) but not different between the 30 and 100 μg/kg doses (p ≥ 0.49).

### 2.8 IgG-(IL-2N88D)_2_ is selective at *in vivo* cell expansion in cynomolgus monkeys

Since our goal was for IgG-(IL-2N88D)_2_ to be highly selective for T_regs_, we quantified its activity on other IL-2 responsive cells. In contrast to the dramatic CD4^+^ T_reg_ expansions, there were no time- or dose-related changes in total CD4^+^ memory effector T cells (Fig. 6E). Within the CD25^+^ subset of CD4^+^ memory effector T cells, there were dose- and time-dependent changes at one and four days (Fig. 6F): with 100 μg/kg there was a 45% drop in cells at one day followed by a transient 48% rebound at day four and return to normal; after 30 μg/kg there was a 26% drop in cells at one day that returned to normal on day four. These changes in the CD25^+^ subset of CD4^+^ memory effector T cells in response to IgG-(IL-2N88D)_2_ were much less than those observed in either CD4^+^ or CD8^+^ T_regs_ (Fig. 4, Fig. 6). No changes were seen in CD8^+^ memory effector T cells or in NK cells other than a drop in cells at one day and return to normal several days later (Supplemental Fig. 7).

While the T_reg_-selective responses to IL-(2N88D)_2_ were maintained at 30 and 100 μg/kg (170 and 570 pmol/kg, respectively), responses to 36 μg/kg (226 pmol/kg) wild-type IgG-IL-2 lost their T_reg_ selectivity and increases in other IL-2 responsive cells such as CD4^+^ and CD8^+^ memory effector T cells and NK cells were now present (Supplemental Fig. 8A–C).

We previously noted an eosinophilia that occurred after several weeks of multiple-dose Proleukin [29]; a known and undesired response in humans and cynomolgus. The highest dose of wild-type IgG-IL-2 tested (36 μg/kg) produced a large expansion in T_regs_ (Fig. 4A) but was accompanied by an eosinophilia in all animals (Fig. 6G). In contrast, 100 μg/kg of IgG-(IL-2N88D)_2_ did not increase cynomolgus' eosinophils despite inducing the largest expansion in T_regs_ (Fig. 4, Fig. 6A,B). There were no dose-related changes observed in other cell types such as neutrophils, monocytes or basophils (not shown).

### 2.9 IgG-(IL-2N88D)_2_ induces T_reg_ specific responses in humanized mice unlike wild-type IL-2

**Fig. 7:**
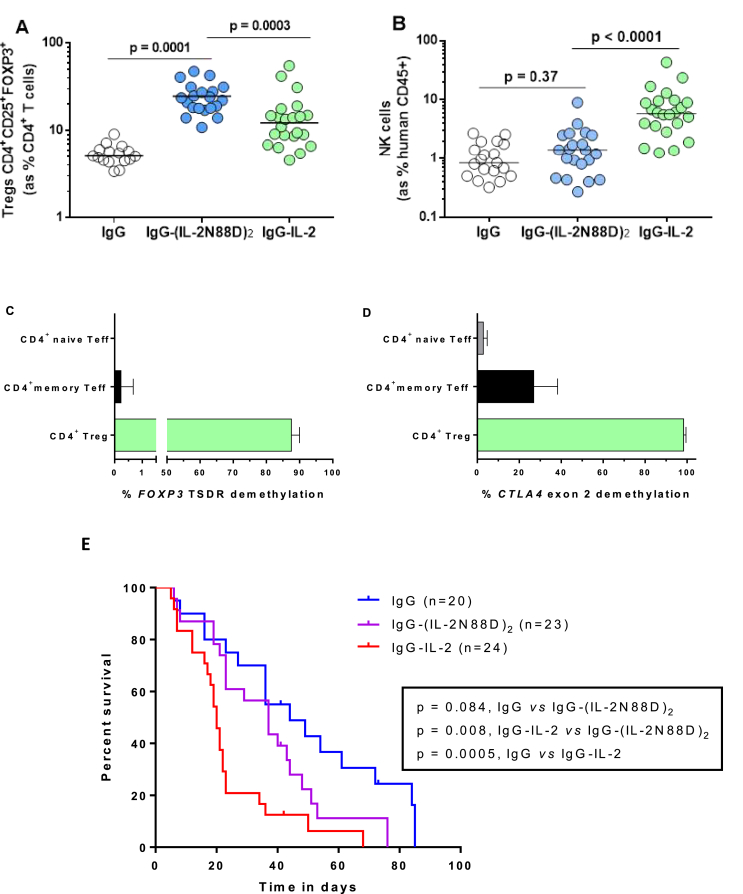
**The *in vivo* effects of IL-2 in humanized mice**. Wild type monomer IgG-IL-2 and IgG-(IL-2N88D)_2_ were tested for their ability to expand human T_regs_ and NK cells. T_regs_ and NK cells were assessed in blood 2 days following the third dose of the indicated molecule using the doses described in the Methods. (**A**) Human CD4^+^CD25^+^FOXP3^+^ T_regs_ are shown as the % of human CD45^+^CD4^+^ cells in blood. (**B**) Human CD3^−^CD16^+^ NK cells in blood are shown as the % of human CD45^+^ cells in blood. Epigenetic signatures were quantified on naïve and memory CD4^+^ effector T cells and CD4^+^ T_regs_ for (**C**) *FOXP3* TSDR in intron 1 and (**D**) *CTLA4* exon 2 as described in Fig. 5. Results for (**C, D**) are shown as the mean ± SD. (**E**) Survival of humanized mice treated with IgG-(IL-2N88D)_2_, IgG-IL-2 and IgG. P values were determined by the log-rank (Mantel-Cox) test (conservative).

While not a replicate of human immune physiology, we rationalized that using humanized mice provided an opportunity to compare wild-type IgG-IL-2 and IgG-(IL-2N88D)_2_ in an *in vivo* setting with human cells as the targeted responders. IgG-IL-2 was chosen as the comparator for IgG-(IL-2N88D)_2_ since they have similar PK properties in NOD-*Prkdc*^*scid*^ *Il2rg*^*null*^ mice (not shown) and share the closest activities on human T_regs_ in whole blood pSTAT5 assays (compare results for IgG-IL-2 in Ref. [29] with those reported for IgG-(IL-2N88D)_2_ in the current study). Wild-type IgG-IL-2 increased CD4^+^ T_regs_ 186% (Fig. 7A) and NK cells 530% (Fig. 7B). In contrast, IgG-(IL-2N88D)_2_ increased CD4^+^ T_regs_ 361% (Fig. 7A) and had no effect on NK cells (Fig. 7B). The increase in T_regs_ following IgG-(IL-2N88D)_2_ was of a significantly greater magnitude and did not overlap with the level of T_regs_ in vehicle-treated mice.

We performed methylation analyses of human *FOXP3* on sorted spleen cells from humanized mice after *in vivo* treatment with IgG-(IL-2N88D)_2_. Newly expanded CD4^+^ T_regs_ were 88% demethylated at the human *FOXP3* TSDR compared to <0.5% of CD4^+^ memory and naïve effector T cells (Fig. 7C). A similar examination of *CTLA4* showed that human CD4^+^ T_regs_ were >98% demethylated while CD4^+^ naïve and memory effector T cells had low to moderate degrees of *CTLA4* demethylation (Fig. 7D). The combination of fully demethylated *FOXP3* and *CTLA4* signatures is a strong indicator of a functionally suppressive phenotype for the *in vivo* expanded human CD4^+^ T_regs_.

We next asked if CD4^+^ T_regs_ from humanized mice were functional after IgG-(IL-2N88D)_2_ treatment by co-culturing T_regs_ with effector T cells stimulated with anti-CD3/anti-CD28 and pre-loaded with an intracellular fluorescent dye to quantify their proliferation by measuring the reduction of fluorescence. After three days of stimulation with anti-CD3/anti-CD28, a majority of effector cells had undergone 1 to 3 cell divisions and the addition of IgG-(IL-2N88D)_2_-expanded T_regs_ suppressed proliferation to that of unstimulated CD4^+^ effector T cells (Supplemental Fig. 9). Finally, we assessed a group of mice treated chronically starting at 3–4 months of age for the influence of IgG-(IL-2N88D)_2_ as compared to IgG-IL-2 on the xenogeneic graft versus host disease that occurs in this humanized mouse model (Fig. 7E). Whereas IgG-(IL-2N88D)_2_ did not increase the rate of disease as compared to vehicle-treated mice, IgG-IL-2 accelerated disease significantly, consistent with our hypothesis that a Treg-selective IL-2 mutein would activate effector T cells and NK cells to a lesser degree than wild type IL-2.

## 3 Discussion

Treating autoimmune diseases with new biologic therapies has seen advances in patient benefit, particularly in rheumatoid arthritis. Whilst these new medicines have unarguably expanded our treatment options, large unmet medical needs persist in all autoimmune diseases. A loss of tolerance and a disruption in immune homeostasis are at the core of autoimmunity and many inflammatory diseases and have been strongly associated with impaired T_reg_ responses, inadequate numbers of T_regs_, as well as effector T cells resistant to suppression [49]. Several clinical approaches to overcome T_reg_ deficiencies are being explored in type 1 diabetes (ClinicalTrials.gov NCT02411253, DIABIL-2) and autoimmune disorders like SLE [43]; most notably adoptive transfer of *in vitro* expanded polyclonal T_regs_ [16] and low-dose Proleukin therapy similar to what has shown patient benefit in chronic GVHD [34,35] and hepatitis C-induced vasculitis [50].

Our first attempt at selectively expanding T_regs_ *in vivo* began with administering low doses of wild-type monomer IgG-IL-2 and wild-type dimer IgG-(IL-2)_2_ to cynomolgus monkeys [29]. These long-lived IL-2 molecules allowed for a sustained *in vivo* increase of T_regs_ following a single dose that achieved a relatively good specificity for T_regs_ insofar that the preferential increase of T_regs_ as compared to eosinophils was superior to that observed with multiple dosing of short-lived Proleukin. However, with higher doses of wild-type IgG-IL-2, not only T_regs_ but NK cells, CD4^+^ and CD8^+^ effector T cells and eosinophils expanded (Supplemental Fig. 8) [29]. These data provided the rationale to design a mutated IL-2 molecule that could be used in patients across a wide dose range to activate and expand T_regs_ more selectively than wild-type IL-2. To accomplish this, we engineered an IL-2 mutein (N88D) with less binding affinity to the predominantly expressed intermediate affinity IL-2Rβγ receptor and made a fusion protein with non-targeted, effector-silent human IgG1 as an *in vivo* half-life enhancer. The optimum stoichiometry was one IgG to two IL-2N88D molecules. In binding studies to the human IL-2Rβγ receptor, IgG-(IL-2N88D)_2_ had significantly lower affinity and avidity than its wild-type counterparts IgG-(IL-2)_2_ and IgG-IL-2.

With this reduced binding affinity, we reasoned IgG-(IL-2N88D)_2_ might be more T_reg_-selective than wild-type IL-2 and this was confirmed in human whole blood pSTAT5 assays. Both the wild type dimer IgG-(IL-2)_2_ and Proleukin stimulated all of the IL-2 responsive cells including CD4^+^ and CD8^+^ memory effector T cells, NK cells and CD56^bright^ NK cells (Fig. 2, Fig. 3). In contrast, IgG-(IL-2N88D)_2_ stimulated memory and naïve T_regs_ and had no effect on other cell types other than some activity on CD56^bright^ NK cells. Its ability to stimulate T_reg_ pSTAT5 was only reduced 6–9-fold compared to Proleukin, in agreement with its 6-fold loss in binding affinity versus monomeric IgG-IL-2.

The penultimate question was how active and selective would IgG-(IL-2N88D)_2_ be *in vivo*. Compared to Proleukin, it was pharmacologically superior and could be administered to cynomolgus monkeys less frequently and expand T_regs_ in a dose-dependent manner to greater extents than Proleukin. In fact, a single low dose expanded CD4 T_regs_ 10-fold with no effect on other cell types. Humanized mice confirmed that IgG-(IL-2N88D)_2_ was more potent and selective for T_reg_ expansion than wild-type IL-2. These *in vivo* activated and expanded cynomolgus and human T_regs_ retained demethylated epigenetic signatures for *FOXP3* and *CTLA4* providing evidence that they would be functionally immunosuppressive. IgG-(IL-2N88D)_2_ also stimulated a population of CD8^+^CD25^+^FOXP3^+^ T_regs_ that is normally present in low numbers. These CD8^+^ T_regs_ were strikingly elevated (14-fold) and had activated biomarker phenotypes for pSTAT5, CD25, Ki-67 and FOXP3. T_reg_-associated *FOXP3* and *CTLA4* epigenetic signatures were observed in expanded CD8^+^FOXP3^+^ sorted cells although not to the same extent as seen in CD4 T_regs_. The potential for therapeutic improvements in bone marrow transplantation and chronic GVHD [36] with an expansion in CD8^+^ T_regs_ makes this an exciting prospect. Although the potential toxicity of IL-2 treatment in patients with type 1 diabetes has been reported, we note very high doses of Proleukin were used which were accompanied by a large induction of NK cells and eosinophils [51]. The interpretation of the study was also further complicated by the concurrent administration of rapamycin with the Proleukin. Nevertheless, these results certainly suggest that dosing levels of Proleukin remain to be optimized and that the development of T_reg_-selective IL-2 muteins such as IgG-(IL-2N88D)_2_ are warranted.

Limitations in our study include not assessing *FOXP3* and *CTLA4* demethylation exclusively in the CD25^hi^ CD8^+^FOXP3^+^ T_regs_ and that NK cells, but not CD56^bright^ NK cells, were monitored in the cynomolgus responses to IgG-(IL-2N88D)_2_. When the epigenetic signatures of *FOXP3* and *CTLA4* in newly expanded CD8^+^ T_regs_ were found to be half that of CD4 T_regs_, a reanalysis found that the CD8^+^ T_regs_ had been sorted based only on FOXP3 expression, not FOXP3 and CD25. This could have led to the inclusion of CD8^+^ Teff expressing FOXP3 in the sorted population. Given their heightened response to IgG-(IL-2N88D)_2_, further analysis of this relatively unstudied CD8^+^ T_reg_ subset will be of interest. At the time of our prior tests we had evaluated a panel of human CD56 staining reagents and found none that were particularly effective or reproducible on cynomolgus NK cells. However, we recently tested a human CD56 reagent listed in the NIH nonhuman primate reagent resource and found similar expression of CD56^bright^ NK cells in cynomolgus as in human (Supplemental Fig. 10). Any further tests will include this reagent in the staining panels so that CD56^bright^ NK cells can be monitored. Another limitation of our study is that due to the immunogenicity of IgG-(IL-2N88D)_2_ in mice, we could not assess its function in any models of autoimmune disease. Multiple dose studies in cynomolgus were also not performed due to the immunogenicity of IgG-(IL-2N88D)_2_ in this species.

In this study, we describe a new IL-2 mutein immunotherapeutic reagent that is T_reg_-specific and pharmacologically superior compared to Proleukin, its clinically available counterpart being used by multiple investigators. With its superior *in vivo* exposure and selective activation properties, IgG-(IL-2N88D)_2_ can expand T_regs_ *in vivo* over a broad dose range without the liability of activating and expanding CD4^+^ and CD8^+^ memory effector T cells, NK cells and eosinophils. This next generation immunotherapy has the potential to correct T_reg_ deficiencies and restore the balance between T_regs_ and effector T cells that is perturbed in autoimmune diseases and other immune-based inflammatory disorders. With IgG-(IL-2N88D)_2_'s wide range of *in vivo* activity, personalized medicine could be attained with each patient's individual needs for restoring immune homeostasis accommodated by dose-dependent adjustments in T_reg_ numbers. Furthermore, as is becoming common in immune system-based therapies in oncology, IgG-(IL-2N88D)_2_ could be used in combination with other therapies to complement each other's activity in unique clinical situations.

Our results in nonhuman primates demonstrate the feasibility of developing a long-lived IL-2 mutein with reduced binding to the intermediate affinity IL-2Rβγ receptor that selectively expands T_reg_ cells *in vivo*. Most notable was the lack of expansion of eosinophils and NK cells by the IL-2 mutein (N88D) at doses where substantial expansion of T_reg_ cells occurred, a specificity not observed with either short- or long-lived wild-type IL-2 molecules that increased eosinophils and NK cells as well as T_reg_ cells. IgG-(IL-2N88D)_2_ treatment of patients with autoimmune diseases should provide benefit due to this preferential expansion of T_reg_ cells that will in turn suppress autoreactive effector T cells.

## 4 Methods

### 4.1 IL-2 molecules

Recombinant human IL-2, Proleukin^®^ (aldesleukin), was obtained from a local pharmacy and the IL-2 fusion proteins, IgG-IL-2, IgG-(IL-2)_2_ and IgG-(IL-2N88D)_2_, were prepared at Hoffman-La Roche [29]. The molecules consist of a human IgG1 with V-domain germline sequences and an engineered short VH CDR3 that has no known antigen-binding properties on human cells or tissues. Specific point mutations in the Fc-portion of the IgG1 (P329G L234A L235A) rendered it effector silent by abolishing C1q and FcRγ binding while leaving normal FcRn function intact [52]. Each IgG1 was engineered to have one or two wild-type or two N88D mutein human IL-2 molecules covalently fused at their N-terminal amino acid to the C-terminus of one or both of the IgG1 heavy chains (omitting the C-terminal lysine) via a flexible (G4S)_3_-peptide linker, *i.e*. IgG-IL-2, IgG-(IL-2)_2_. Knobs-into-holes technology was used to engineer monovalent IgG-IL-2 [53]. The molecular weights used to calculate pM and pmoles/kg are as follows: IgG-IL-2 (159.1 kDa), IgG-(IL-2)_2_ (175.3 kDa) and IgG-(IL-2N88D)_2_ (175.3 kDa) and Proleukin (15.3 kDa).

### 4.2 Surface plasmon resonance

Binding affinities were measured by surface plasmon resonance on human, cynomolgus and murine IL-2Rα and IL-2Rβγ on a Biacore T200 (GE Healthcare). Monomeric his-tagged IL2Rα was chemically immobilized by amine coupling on CM5 chips. A 2-fold dilution series (0.41 nM–300 nM) of IgG-IL-2 and IgG-(IL-2)_2_ was injected over the chip surface for 90 seconds and the dissociation was monitored for 3 minutes. The surface was regenerated after each injection by washing with 10 mM glycine pH 1.5 for 60 seconds. Due to the fast association and dissociation rates, the binding curves were fitted using a steady state model (BIAevaluation software). To measure binding to IL2Rβγ, a heterodimeric Fc fusion was generated by applying knobs-into-holes technology [53]. The IL2Rβ and γ chains were fused to the hole or knob chain of the Fc, respectively, and co-expressed to obtain the preformed recombinant heterodimer also carrying an avi-tag for site-specific biotinylation. These biotinylated IL2Rβγ heterodimers were immobilized on streptavidin chips. A 2-fold dilution series of the cytokine fusion constructs (1.2 nM–300 nM) was injected over the chip surface for 2 minutes and the dissociation was monitored for 10 minutes (for the two highest concentrations) to observe a measurable decay of these high-affinity complexes. The surface was regenerated after each injection by washing with 3 M MgCl_2_. The binding curves were fitted globally using a 1:1 interaction model (BIAevaluation software) despite the bivalent binding of IgG-(IL-2N88D)_2_ and IgG-(IL-2)_2_ resulting in an ‘apparent’ K_D_. The very slow off-rates of IgG-(IL-2)_2_ on human IL2Rβγ results in a low K_D_ in the single-digit pM range that approaches the limit of the instrument.

### 4.3 In vitro pSTAT5 activation in human and cynomolgus whole blood

Human blood from healthy adults was collected with informed consent from the Cambridge BioResource with ethical approval by the Peterborough and Fenland Local Research Ethics Committee (05/Q0106/20). The effects of Proleukin, IgG-(IL-2)_2_ and IgG-(IL-2N88D)_2_ on the induction of pSTAT5 were assessed in human naïve and memory CD4^+^ T_reg_ subsets, naïve and memory conventional CD4^+^ T cells, naïve and memory conventional CD8^+^ T cells, NKT cells and NK cells as previously described [29]. The antibodies used for staining human cells are shown in Supplemental Table 3. Blood from healthy cynomolgus monkeys was stimulated with IgG-(IL-2)_2_ and IgG-(IL-2N88D)_2_ and pSTAT5 induction was assessed similarly to that with human blood. The antibodies used for staining cynomolgus blood cells are shown in Supplemental Table 4.

### 4.4 Cynomolgus monkeys

All cynomolgus monkeys used to test the various IL-2 fusion proteins had never received a human protein (Charles River Laboratories, Edinburgh, Scotland and Hoffmann-La Roche, Nutley, NJ). All procedures were performed with adherence to the NIH Guide for the Care and Use of Laboratory Animals and were approved by the Roche Institutional Animal Care and Use Committee and the Roche Ethics Committee for Animal Welfare. Individual subcutaneous doses were based on body weight and were formulated in 25 mM Citrate, 300 mM Arginine, pH6.7 with 0.5% normal cynomolgus serum. Arginine is included in the buffer to reduce the likelihood of aggregation [54]. The safety, efficacy and biologic responses to treatments were monitored in all blood samples taken during the studies using clinical hematology and chemistry analyses. Lymphocyte and eosinophil numbers were obtained from the clinical hematology complete blood count and, in conjunction with flow cytometry data, were used to calculate absolute numbers (cells/mm^3^ or cells/ml) of specific cell subsets.

### 4.5 Effects of IL-2 *in vivo* in humanized mice

The research involving mice in this study has been regulated under the Animals (Scientific Procedures) Act 1986 Amendment Regulations 2012 following ethical review by the University of Cambridge Animal Welfare and Ethical Review Body (AWERB). Humanized mice were constructed using NOD-*Prkdc*^*scid*^ *Il2rg*^*null*^ (NSG) mice as hosts and engrafting them with human fetal liver CD34^+^ stem cells. For the experiment assessing demethylation of the TSDR (Fig. 7) in T cell subsets, we used mice constructed with male stem cells since *FOXP3* is on the X chromosome and due to X inactivation, demethylation in females is only present on one of the two X chromosomes, thereby lowering the sensitivity of the measurement. On the first or second day of birth, neonatal NSG mice received 100 rads of whole-body irradiation and were injected with CD34^+^ stem cells isolated from human fetal liver obtained from Advanced Bioscience Resources, Inc., Alameda, CA, USA; each mouse received 0.25-0.5 × 10^6^ cells by intrahepatic injection. At 10 weeks of age, mice were assessed for human CD45^+^ cell engraftment present in blood. Mice were not used in dosing experiments unless more than 5000 human CD45^+^ cells per ml of blood were present and at least 8% of the blood cells were CD3^+^ T cells. T_regs_ were CD45^+^CD3^+^CD4^+^FOXP3^+^CD25^+^CD127^lo^ and NK cells were CD45^+^CD3^−^CD16^+^. For dosing, the IL-2 fusion proteins were formulated in 25 mM Citrate, 300 mM Arginine, pH6.7 with 0.5% normal serum to prevent binding loss to tubes and syringes. At 3–4 months of age, mice matched for human cell reconstitution and age across treatment groups were injected subcutaneously with 4 pmoles (0.7 μg) of the IL-2 fusion molecules or human IgG twice weekly (Monday/Thursday or Tuesday/Friday). The antibodies for staining cells from humanized mice are in Supplemental Table 5.

### 4.6 T_reg_ suppression assays

Using humanized mice, the functional activity of T_regs_ expanded *in vivo* by IgG-(IL-2N88D)_2_ was determined in an *in vitro* co-culture assay measuring the ability of T_regs_ to suppress the proliferation of CD4^+^ memory effector T cells stimulated by anti-CD3 and anti-CD28. Mice were treated with IgG-(IL-2N88D)_2_ and spleen cells were sorted into T_regs_ (CD45^+^CD3^+^CD4^+^CD25^+^CD127^lo^) and memory effector T cells (CD45^+^CD45RA^−^CD3^+^CD4^+^CD25^+/-^CD127^hi^). Prior to sorting, the effector T cells were stained with the cell proliferation dye eFluor 450 (eBioscience) according to the manufacturer's instructions. Memory effector T cells were cultured for three days at 5 × 10^4^ cells in U-bottom plates and stimulated with plate-bound anti-CD3 (1 μg/ml) and soluble anti-CD28 (2 μg/ml); T_regs_ were added to the effector cells at a 1:1 ratio. Effector T cells stained with proliferation dye eFluor 450 were assessed for cell division using flow cytometry on a LSRFortessa (BD Biosciences) and analyzed with FlowJo software.

### 4.7 DNA demethylation of FOXP3 and CTLA4

The epigenetic methylation signatures of *FOXP3* and *CTLA4* were tested in sorted CD4^+^ and CD8^+^ T cell subsets from cynomolgus monkeys and in sorted human CD4^+^ T cell subsets from humanized mice after treatment with IgG-(IL-2N88D)_2_. All of the cynomolgus monkeys were male to increase the sensitivity of the measurement as detailed above. The details for preparing and testing the DNA methylation status of cynomolgus and human *FOXP*3 and *CTLA4* have been described [28,29]. In brief, we used next-generation sequencing (NGS) of bisulfite-treated DNA and quantified the methylation status of the *FOXP3* TSDR (Treg-specific demethylated region). In humans, the *FOXP3* TSDR has 9 CpG sites in intron 1 and in cynomolgus the *FOXP3* TSDR contains 10 CpG sites in intron 1. Similarly, for *CTLA4*, we quantified the 7 and 9 CpG sites in exon 2 in human and cynomolgus, respectively. This method reports single base resolution, C (methylated) or T (demethylated), for each CpG site and assesses thousands of reads per replicate. Human CD4^+^FOXP3^+^ Tregs were classified by ≥ 8 of the 9 CpG sites demethylated and cynomolgus CD4^+^FOXP3^+^ Tregs had ≥9 of the 10 CpG sites demethylated. Sorted human and cynomolgus naïve and memory CD4^+^ effector T cells had the opposite pattern with >95% of the reads having ≤1 CpG sites demethylated.

### 4.8 Data analysis

Prism 6 (GraphPad Software, Inc) was used to calculate means and standard deviations as well as the differences between survival curves.

## Author contributions

LBP, LSW, KB and RJH participated in the design of the experiments and interpretation of results. LBP, LSW, SKH, CJMB, MLP, DS, HH, AF-G and RJH participated in the acquisition and analysis of data. EM, CK, RJH, PU, LBP and LSW designed the immunocytokines. JAT facilitated the use of humanized mice. OA generated the immunocytokines. IW and AF-G characterized the immunocytokines. LBP wrote the manuscript and all of the authors reviewed and commented on the manuscript.

## Competing interests

KB, OA, DS, HH, PU, IW, AF-G, EM, CK and RJH are shareholders in Roche.
