# Supplementary material for: A long-lived IL-2 mutein that selectively activates and expands regulatory T cells as a therapy for autoimmune disease
Source: J Autoimmun. 2018 Dec;95:1–14. doi: 10.1016/j.jaut.2018.10.017 (PMC6284106; doi:10.1016/j.jaut.2018.10.017)
Supplement: Multimedia component 1 [file mmc1.docx]

**SUPPLEMENTAL FIGURES**


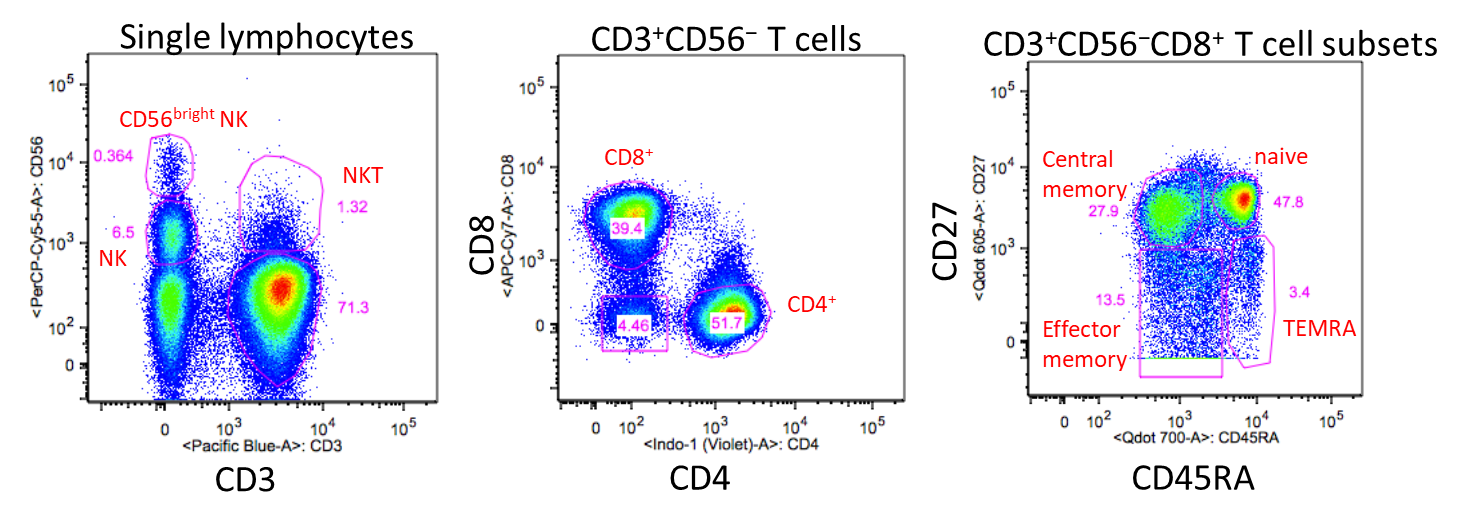


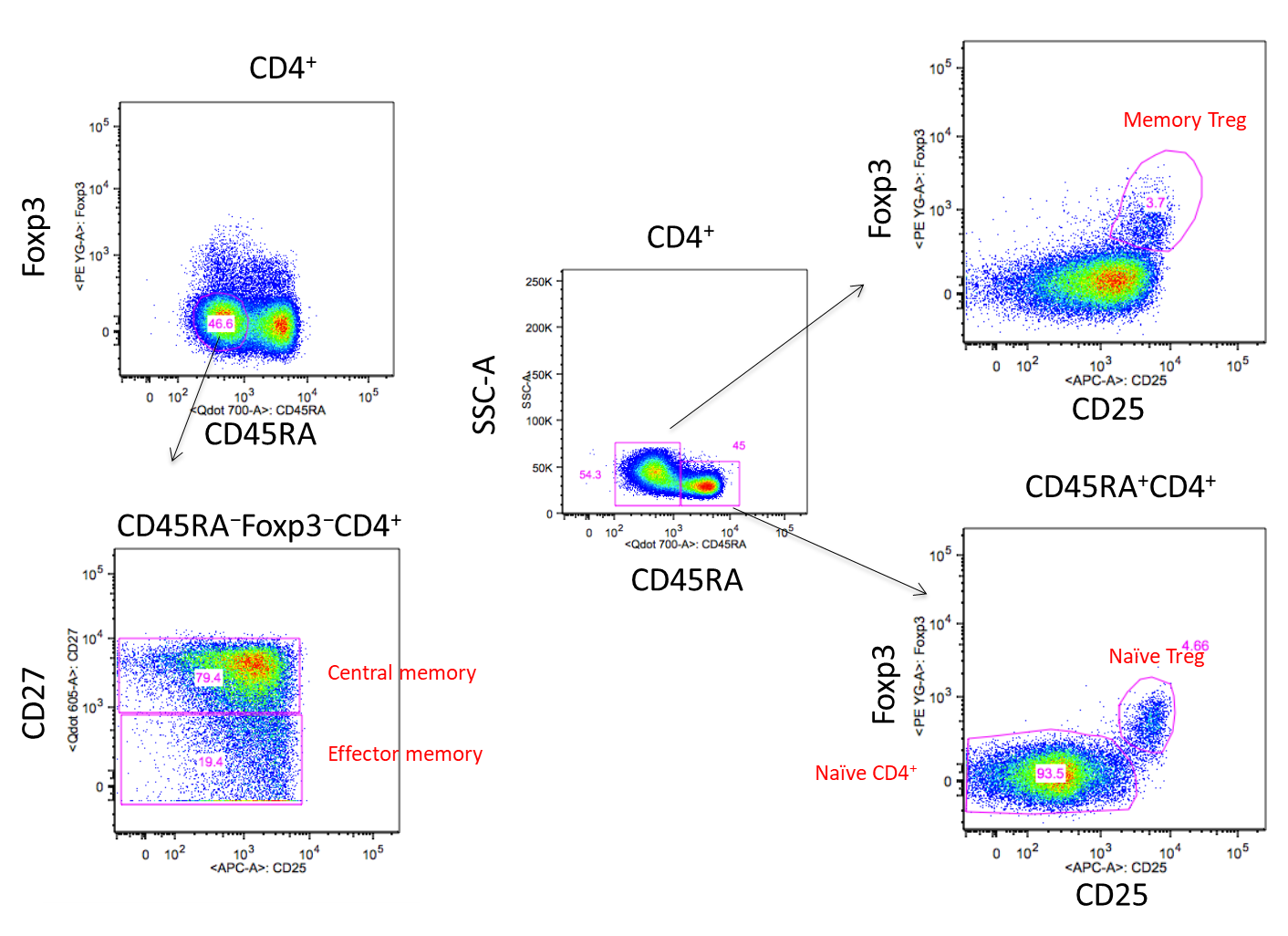


**Supplemental Fig 1. Gating strategy for cell subsets in the human whole blood pSTAT5 IL-2 activation assay shown in Figure 2.** Details of the antibodies used to stain cell subsets described above are shown in Supplemental Table 3.

**
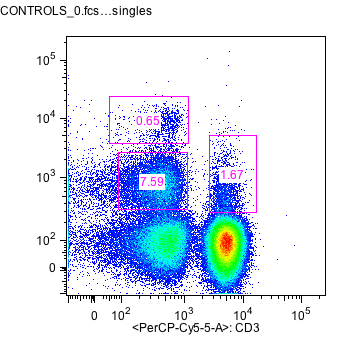
**

CD56^bright^ NK cells

NK T cells

CD56

NK cells


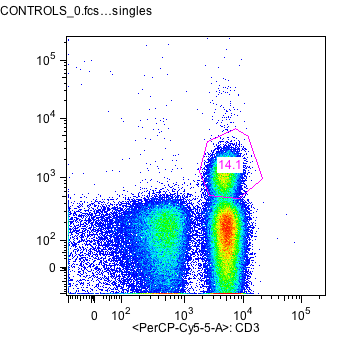

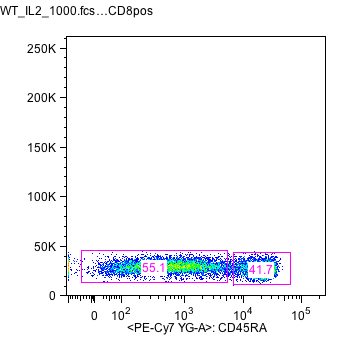


CD3

Memory CD8^+^ T cells

CD45RA^+^ CD8^+^ T cells

(naïve and TEMRA)

SSC

CD8

CD3

CD45RA

**
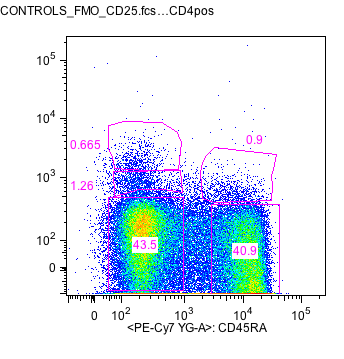
**

Memory FOXP3^high^ Tregs

Memory FOXP3^low^ Tregs

FoxP3

Naive Tregs

CD45RA

Memory CD4^+^ T cells

Naive CD4^+^ T cells

**Supplemental Fig 2. Gating strategy for cell subsets in the human whole blood pSTAT5 IL-2 activation assay shown in Figure 3.** Details of the antibodies used to stain cell subsets are shown in Supplemental Table 3.

**Supplemental Fig 3. Cynomolgus whole blood pSTAT5 responses.** IgG-(IL-2N88D)_2_ and wild-type IgG-(IL-2)_2_ were tested for their abilities to induce pSTAT5 in cynomolgus whole blood. Subsets of CD4^+^ T_regs_ and CD4^+^ memory effector T cells were tested for responsiveness. Shown are results from one animal.


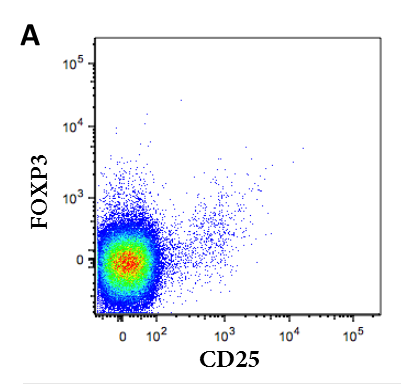

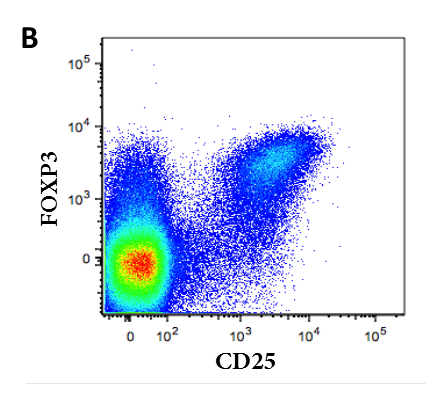


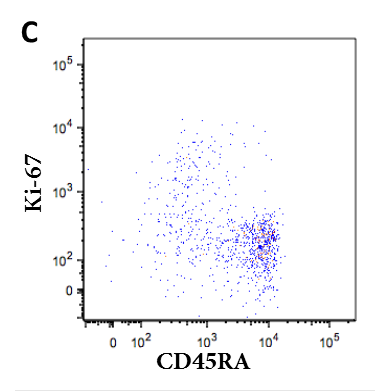

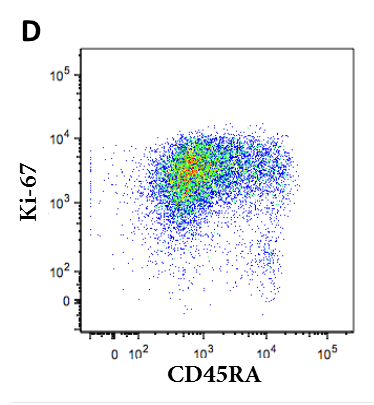


**Supplemental Fig 4. CD8^+^ T_regs_ increase and change phenotype.** (**A**) Expression of FOXP3 and CD25 in normal cynomolgus CD8^+^ T cells. (**B**) FOXP3 and CD25 expression in CD8^+^ T cells four days after 100 µg/kg IgG-(IL-2N88D)_2_. (**C**) Before stimulation with IgG-(IL-2N88D)_2_, cynomolgus CD8^+^CD25^hi^ FOXP3^+^ T_regs_ were in low abundance and the majority were CD45RA^+^ and Ki-67^−^, a naïve phenotype. (**D**) After stimulation with IgG-(IL-2N88D)_2_, CD8^+^CD25^hi^ FOXP3^+^ T_regs_ expanded greatly and the majority now expressed a memory phenotype (CD45RA^−^) and were in cell cycle, (Ki-67^+^).

**
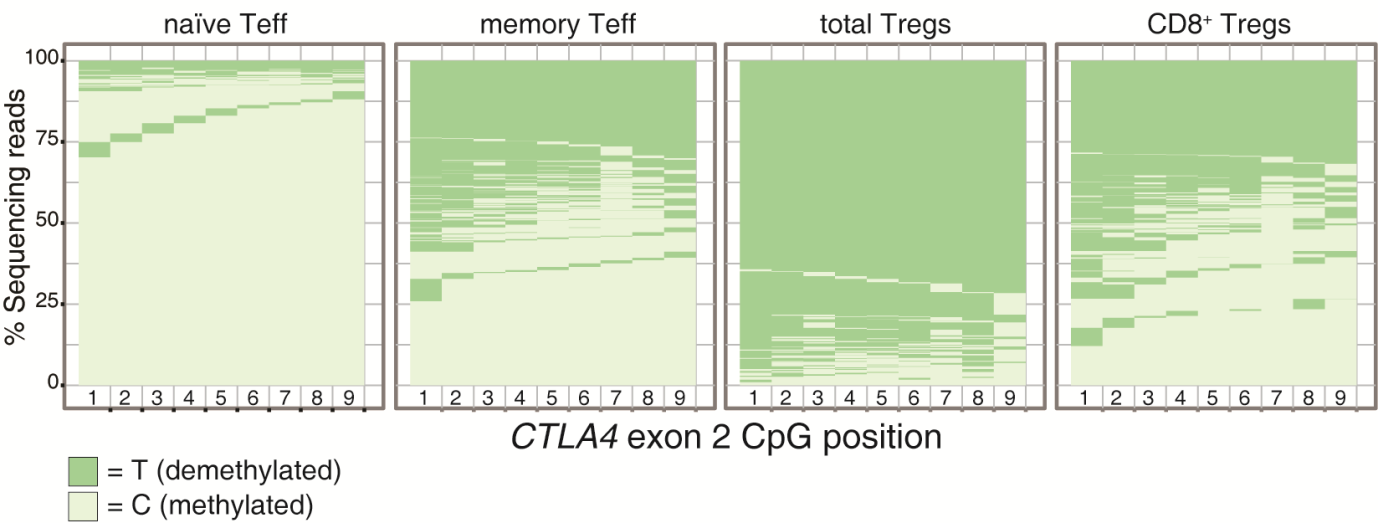
**

**Supplemental Fig 5. Cynomolgus epigenetic methylation signatures of *CTLA4*.** Methylation signatures for *CTLA4* exon 2 in sorted CD4^+^ T cell subsets and CD8^+^ Tregs after *in vivo* expansion with 100 µg/kg IgG-(IL-2N88D)_2_. Visual representations of the methylated (C, cytosine) and demethylated (T, thymine) sequencing reads at each of the nine CpG sites for *CTLA4*.

**Supplemental Fig 6. *Ex vivo* evaluation of cynomolgus biomarkers in non-T_reg_ cells.** Time-dependent changes in IgG-(IL-2N88D)_2_-induced: (**A**) pSTAT5, (**B**) CD25 and (**C**) Ki-67; 100 µg/kg, n=4, mean ± SD. The y-axes were set to the ranges that were used to accommodate IgG-(IL-2N88D)_2_-induced T_reg_ responses, thus highlighting the unresponsive nature of the T_conv_ cells analyzed here.

**Supplemental Fig 7. Changes in cynomolgus NK cells and CD8^+^ T cells.** Time-dependent changes in NK cells and CD8^+^ memory effector T cells before and after a single dose of IgG-(IL-2N88D)_2_; 100 µg/kg, n=4, mean ± SD.

**Supplemental Fig 8. Cynomolgus responses to high dose IgG-IL-2.** With an IgG-IL-2 dose of 36 µg/kg, *in vivo* responses were no longer T_reg_-specific and additional IL-2-responsive cells expanded *in vivo*: (**A**) CD4^+^ memory effector T cells, (**B**) CD8^+^ memory effector T cells and (**C**) CD3^−^CD16^+^ NK cells; all results are from n=5, mean ± SD.

**
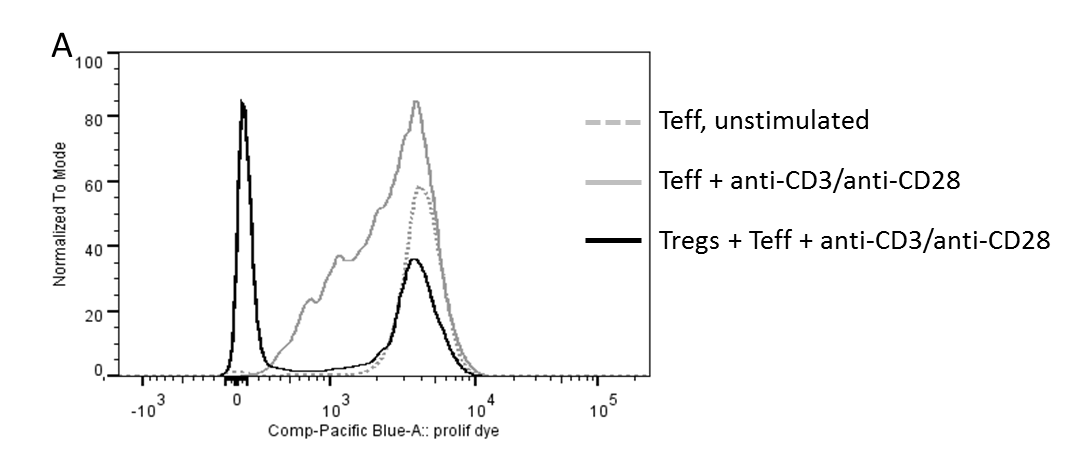
**

**Supplemental Fig 9. T_reg_ functional activity in humanized mice.** (**A**) A co-culture suppression assay with IgG-(IL-2N88D)_2_ *in vivo*-expanded human CD4 T_regs_ at a 1:1 ratio with *in vitro* anti-CD3/anti-CD28 stimulated CD4 effector T cells. CD4 T_reg_ proliferation was quantified after three days by a preloaded fluorescent dye (representative of three different tests). CD4 T_regs_ did not have fluorescent dye loaded and are seen as the unstained peak on the left at 0.


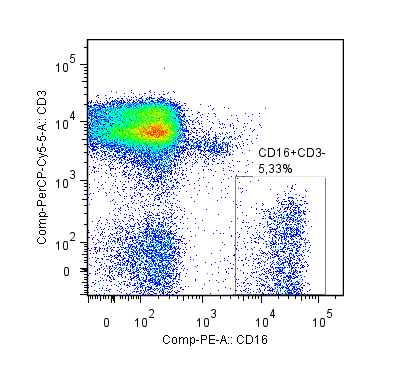

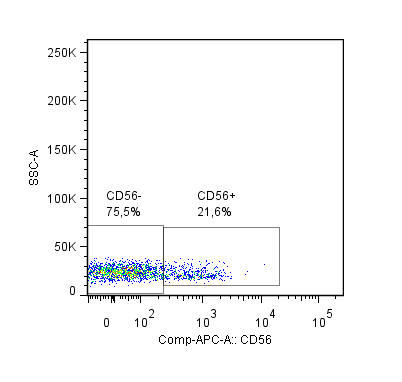


**Supplemental Fig 10. Cynomolgus NK cells express CD56.** Blood from a normal cynomolgus monkey was tested for the expression of CD56 on NK cells (CD3^−^CD16^+^) by flow cytometry. Clone MY31, BD Biosciences was used to stain cynomolgus NK cells.

| **Human** | **IL-2Rα** | **K_D_ steady state (nM)** |
| --- | --- | --- |
| IgG-IL-2 | Human | 15 |
|  | Cynomolgus | 30 |
| IgG-(IL-2)_2_ | Human | 5 |
|  | Cynomolgus | 15 |
| IgG-(IL-2N88D)_2_ | Human  Cynomolgus | 22  30 |

**Supplemental Table 1. Binding of human IL-2 fusion proteins to the IL-2Rα receptor.** The binding affinities (K_D_) for three different IL-2 fusion proteins were measured by surface plasmon resonance on a BIACORE T200 performing steady state analysis.

| **CYNOMOLGUS** | **IgG-(IL-2)_2_**  **EC_50_ (pM)** | **IgG-(IL-2N88D)_2_**  **EC_50_ (pM)** | **Fold-change**  **in EC_50_** |
| --- | --- | --- | --- |
| memory T_regs_ | 0.7 | 14 | **14-fold** |
| naïve T_regs_ | 0.3 | 4 | **13-fold** |

**Supplemental Table 2. EC_50_ results of cynomolgus whole blood pSTAT5 assays.** Blood was stimulated across a wide concentration of IgG-(IL-2)_2_ and IgG-(IL-2N88D)_2_ and pSTAT5 measured. EC_50_s were determined from the dose response curves.

|  | **Clone(s)** | **Panel 1 (Fig. 2)** | **Supplier** | **Panel 2 (Fig. 3)** | **Supplier** |  |
| --- | --- | --- | --- | --- | --- | --- |
| CD25 | 2A3 + M-A251 | APC | BD Biosciences | APC | BD Biosciences |  |
| CD4 | RPA-T4 | BUV395 | BD Biosciences | AF700 | BioLegend |  |
| CD45RA | HI100 | AF700 | BioLegend | PE/Cy7 | BioLegend |  |
| CD8 | RPA-T8 | APC/Cy7 | BioLegend | BV605 | BioLegend |  |
| CD56 | HCD56 | PerCP/Cy5.5 | BioLegend | BV421 | BioLegend |  |
| FOXP3 | 259D | PE | BioLegend | PE | BioLegend |  |
| pSTAT5a | 47/Stat5(pY694) | AF488 | BD Biosciences | AF488 | BD Biosciences |  |
| CD3 | UCHT1 | Brilliant Violet 421 | BioLegend | PerCP/Cy5.5 | BioLegend |  |
| CD27 | L128 | BV605 | BD Biosciences | not used |  |  |

**Supplemental Table 3. Antibodies used for staining human blood cells in Figures 2 and 3.**

| CD3 | APC-Cy-7 | SP34-2 | BD Biosciences |
| --- | --- | --- | --- |
| CD4 | PerCP-Cy-5.5 | L200 | BD Biosciences |
| CD8 | BUV395 | RPA-T8 | BD Biosciences |
| CD25 | PE | 4E3 | eBioscience |
| CD45RA | V450 | 5H9 | BD Biosciences |
| FOXP3 | AF647 | 259D | BioLegend |
| pSTAT5a | AF488 | 47 | BD Biosciences |
| Ki-67 | PE-Cy-7 | B56 | BD Biosciences |
| CD56* | APC | MY31 | BD Biosciences |

**Supplemental Table 4. Antibodies used for staining cynomolgus blood cells. ***CD56 was tested after the in-life experiments were complete.

| CD3 | BV605 | OKT3 | BioLegend |
| --- | --- | --- | --- |
| CD4 | AF700 | RPA-T4 | BioLegend |
| CD8 | APC/Cy7 | RPA-T8 | BioLegend |
| CD25 | APC | 2a3 + M-A251 | BD Biosciences |
| CD45 | BUV395 | HI30 | BD Horizon |
| CD45RA | PB, FITC, or BV785 | HI100 | BioLegend |
| CD56 | PE or PE/Cy7 | HCD56 | BioLegend |
| CD127 | PE/Cy7 | eBioRDR5 | eBioscience |
| CD19 | PE or APC/Cy7 | HIB19 | BioLegend |
| FOXP3 | PE | 259D | BioLegend |

**Supplemental Table 5. Antibodies used for staining human cells from humanized mice.**
